# 3,3′-Dimethyl-1,1′-(butane-1,4-di­yl)diimidazolium bis­(tetra­fluoro­borate)

**DOI:** 10.1107/S160053681001593X

**Published:** 2010-05-08

**Authors:** Hao Geng, Ling-hua Zhuang, Jian Zhang, Guo-wei Wang, Ai-lin Yuan

**Affiliations:** a Department of Light Chemical Engineering, College of Food Science and Light Engineering, Nanjing University of Technology, Nanjing 210009, People’s Republic of China; b Department of Applied Chemistry, College of Science, Nanjing University of Technology, Nanjing 210009, People’s Republic of China

## Abstract

The title compound, C_12_H_20_N_4_^2+^·2BF_4_^−^, was prepared by the anion exchange of a dibromide ionic liquid with sodium tetra­fluoro­borate. The asymmetric unit contains one half of the imidazolium cation, which lies about an inversion centre at the mid-point of the central C—C bond of the linking butyl chain. The two planar imidazole rings (r.m.s. deviation = 0.0013 Å) are strictly parallel and separated by 2.625 (7) Å [vertical distance between the centroids of two imidazole rings], giving the mol­ecule a stepped appearance. In the crystal structure, inter­molecular C—H⋯F hydrogen bonds link the cations and anions, generating a three-dimensional network.

## Related literature

For properties and applications of ionic liquids, see: Welton (1999 ▶); Olivier & Magna (2002 ▶); Nicholas *et al.* (2004 ▶); Yu *et al.*(2007 ▶). For dicationic ionic liquids, see: Leclercq *et al.* (2007 ▶); Payagala *et al.* (2007 ▶). For bond-length data, see: Allen *et al.* (1987 ▶). 
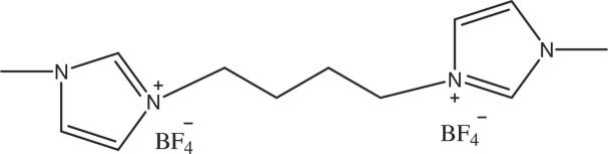


## Experimental

### 

#### Crystal data

C_12_H_20_N_4_^2+^·2BF_4_^−^
*M* *_r_* = 393.94
Monoclinic, 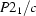

*a* = 5.195 (1) Å
*b* = 14.836 (3) Å
*c* = 11.790 (2) Å
β = 99.53 (3)°
*V* = 896.2 (3) Å^3^
*Z* = 2
Mo *K*α radiation
μ = 0.15 mm^−1^
*T* = 293 K
0.30 × 0.10 × 0.10 mm

#### Data collection

Enraf–Nonius CAD-4 diffractometer
Absorption correction: ψ scan (North *et al.*, 1968 ▶) *T*_min_ = 0.958, *T*_max_ = 0.986
1960 measured reflections
1763 independent reflections
1125 reflections with *I* > 2σ(*I*)
*R*_int_ = 0.019
3 standard reflections every 200 reflections  intensity decay: 1%

#### Refinement

*R*[*F* ^2^ > 2σ(*F* ^2^)] = 0.046
*wR*(*F* ^2^) = 0.155
*S* = 1.01
1763 reflections
119 parameters
H-atom parameters constrained
Δρ_max_ = 0.20 e Å^−3^
Δρ_min_ = −0.19 e Å^−3^

### 

Data collection: *CAD-4 Software* (Enraf–Nonius, 1989 ▶); cell refinement: *CAD-4 Software*; data reduction: *XCAD4* (Harms & Wocadlo, 1995 ▶); program(s) used to solve structure: *SHELXS97* (Sheldrick, 2008 ▶); program(s) used to refine structure: *SHELXL97* (Sheldrick, 2008 ▶); molecular graphics: *SHELXTL* (Sheldrick, 2008 ▶); software used to prepare material for publication: *SHELXTL*.

## Supplementary Material

Crystal structure: contains datablocks global, I. DOI: 10.1107/S160053681001593X/sj2792sup1.cif

Structure factors: contains datablocks I. DOI: 10.1107/S160053681001593X/sj2792Isup2.hkl

Additional supplementary materials:  crystallographic information; 3D view; checkCIF report

## Figures and Tables

**Table 1:** Hydrogen-bond geometry (Å, °)

*D*—H⋯*A*	*D*—H	H⋯*A*	*D*⋯*A*	*D*—H⋯*A*
C2—H2*A*⋯F1^i^	0.93	2.50	3.328 (3)	149
C3—H3*A*⋯F3^ii^	0.93	2.51	3.398 (3)	161
C4—H4*A*⋯F2^iii^	0.93	2.46	3.272 (3)	146
C4—H4*A*⋯F3^iii^	0.93	2.45	3.326 (3)	158

Symmetry codes: (i) 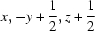; (ii) 
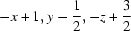
; (iii) 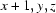.

**Fig. 1.**
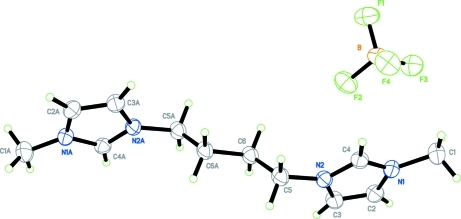
A view of the molecular structure of (I) showing the atom-numbering scheme and 30 % displacement ellipsoids .

**Fig. 2.**
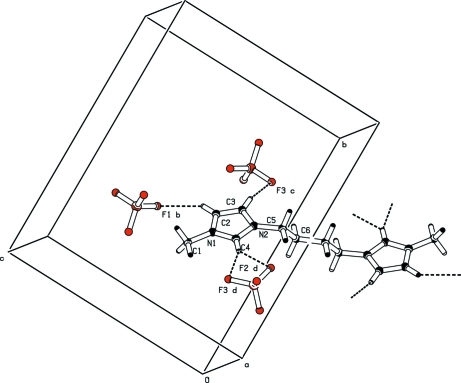
The crystal packing of (I), Hydrogen bonds are drawn as dashed lines. Symmetry codes a) x,1/2-y,1/2+z; b) 1-x,-1/2+y,3/2-z; c) 1+x,y,z

## supplementary crystallographic information

### Comment

Ionic liquids (ILs) are generally formed by an organic cation and a weakly coordinating anion. They have enjoyed considerable research interest in recent years because of their unique properties such as high thermal stability, non-volatility, non-flammability, high ionic conductivity, a wide electrochemical window and miscibility with organic compounds (Welton, 1999; Nicholas *et al.*, 2004; Yu *et al.*, 2007). ILs have been widely applied to several areas including catalysis, electrochemistry, separation science, as solvents for green chemistry, biology and materials for optoelectronic applications (Olivier & Magna, 2002). Geminal dicationic ionic liquids have been shown to possess superior physical properties in terms of thermal stability and volatility compared to traditional ionic liquids (ILs) (Leclercq *et al.*, 2007; Payagala *et al.*, 2007) .

We here report the crystal structure of the title compound (I).

The atom-numbering scheme of (I) is shown in Fig.1, and all bond lengths are within normal ranges (Allen *et al.*, 1987).

The imidazole ring (C2/C3/N2/C4/N1) is planar, with r.m.s. deviation 0.0013 Å. The two imidazole rings are strictly parallel.

In the crystal structure intermolecular C—H···F hydrogen bonds link the cations and anions generating a three-dimensional network. (Table 1 and Fig.2). ).

### Experimental

A solution of 1,4-dibromobutane(4.3 g, 0.02 mol) in methanol(20 ml) was slowly added to a solution of 1-methylimidazole(3.28 g, 0.04 mol) in methanol(20 ml) at room temperature. The reaction mixture was then refluxed for 6 h. After evaporation of the solvent, the residue was washed with diethyl ether and dichloromethane, then dried in vacuum to obtain ionic liquid 3-methyl-1-[4-(1-methylimidazolium-3-yl) butyl]-imidazolium dibromide (a white solid ionic liquid).

A solution of above mentioned dibromide ionic liquid (3.8 g, 0.01 mol) in methanol(20 ml) was slowly added to a solution of sodium tetrafluoroborate (2.2 g, 0.02 mol) in methanol (20 ml), The reaction mixture was refluxed for 1 h. After evaporation of the solvent, the residue was washed with diethyl ether, then dried in vacuum to obtain title compound (I), 3-methyl-1-[4-(1-methylimidazolium-3-yl) butyl]- imidazolium ditetrafluoroborate(yield 94%). M.p. 95-97

Crystals of (I) suitable for X-ray diffraction were obtained by slow evaporation of a methanol solution. ^1^H NMR (D_2_O, δ, p.p.m.) 8.67 (s, 2 H), 7.43 (d, 4 H), 4.23 (s, 4 H), 3.87 (s,6 H), 1.88 (s, 4 H).

### Refinement

All H atoms were positioned geometrically, with C—H = 0.93 Å, and constrained to ride on their parent atoms, with U_iso_(H) = xU_eq_(C), where x= 1.5 for methyl H and x = 1.2 for methylene H atoms.

### Crystal data

**Table:**

C_12_H_20_N_4_^2+^·2BF_4_^−^	*F*(000) = 404
*M**_r_* = 393.94	*D*_x_ = 1.460 Mg m^−^^3^
Monoclinic, *P*2_1_/*c*	Mo *K*α radiation, λ = 0.71073 Å
Hall symbol: -P 2ybc	Cell parameters from 25 reflections
*a* = 5.195 (1) Å	θ = 9–13°
*b* = 14.836 (3) Å	µ = 0.15 mm^−^^1^
*c* = 11.790 (2) Å	*T* = 293 K
β = 99.53 (3)°	Block, colorless
*V* = 896.2 (3) Å^3^	0.30 × 0.10 × 0.10 mm
*Z* = 2	

### Data collection

**Table:**

Enraf–Nonius CAD-4 diffractometer	1125 reflections with *I* > 2σ(*I*)
Radiation source: fine-focus sealed tube	*R*_int_ = 0.019
graphite	θ_max_ = 26.0°, θ_min_ = 2.2°
ω/2θ scans	*h* = 0→6
Absorption correction: ψ scan (North *et al.*, 1968)	*k* = 0→18
*T*_min_ = 0.958, *T*_max_ = 0.986	*l* = −14→14
1960 measured reflections	3 standard reflections every 200 reflections
1763 independent reflections	intensity decay: 1%

### Refinement

**Table:**

Refinement on *F*^2^	Secondary atom site location: difference Fourier map
Least-squares matrix: full	Hydrogen site location: inferred from neighbouring sites
*R*[*F*^2^ > 2σ(*F*^2^)] = 0.046	H-atom parameters constrained
*wR*(*F*^2^) = 0.155	*w* = 1/[σ^2^(*F*_o_^2^) + (0.085*P*)^2^] where *P* = (*F*_o_^2^ + 2*F*_c_^2^)/3
*S* = 1.00	(Δ/σ)_max_ < 0.001
1763 reflections	Δρ_max_ = 0.20 e Å^−^^3^
119 parameters	Δρ_min_ = −0.19 e Å^−^^3^
0 restraints	Extinction correction: *SHELXL97* (Sheldrick, 2008), Fc^*^=kFc[1+0.001xFc^2^λ^3^/sin(2θ)]^-1/4^
Primary atom site location: structure-invariant direct methods	Extinction coefficient: 0.105 (11)

### Special details

**Table:**

Geometry. All esds (except the esd in the dihedral angle between two l.s. planes) are estimated using the full covariance matrix. The cell esds are taken into account individually in the estimation of esds in distances, angles and torsion angles; correlations between esds in cell parameters are only used when they are defined by crystal symmetry. An approximate (isotropic) treatment of cell esds is used for estimating esds involving l.s. planes.
Refinement. Refinement of *F*^2^ against ALL reflections. The weighted *R*-factor wR and goodness of fit *S* are based on *F*^2^, conventional *R*-factors *R* are based on *F*, with *F* set to zero for negative *F*^2^. The threshold expression of *F*^2^ > σ(*F*^2^) is used only for calculating *R*-factors(gt) etc. and is not relevant to the choice of reflections for refinement. *R*-factors based on *F*^2^ are statistically about twice as large as those based on *F*, and *R*- factors based on ALL data will be even larger.

### Fractional atomic coordinates and isotropic or equivalent isotropic displacement parameters (Å^2^)

**Table:**

	*x*	*y*	*z*	*U*_iso_*/*U*_eq_	
N1	0.9683 (4)	0.16123 (13)	0.88401 (17)	0.0515 (6)	
C1	0.9656 (7)	0.24171 (19)	0.9557 (2)	0.0741 (9)	
H1A	1.0716	0.2877	0.9296	0.111*	
H1B	1.0336	0.2269	1.0343	0.111*	
H1C	0.7897	0.2632	0.9504	0.111*	
N2	1.0555 (4)	0.07197 (13)	0.75168 (16)	0.0469 (5)	
C2	0.8390 (5)	0.08211 (17)	0.8951 (2)	0.0569 (7)	
H2A	0.7331	0.0695	0.9494	0.068*	
C3	0.8937 (5)	0.02637 (17)	0.8132 (2)	0.0549 (7)	
H3A	0.8334	−0.0324	0.8002	0.066*	
C4	1.0970 (5)	0.15315 (16)	0.7964 (2)	0.0488 (6)	
H4A	1.1997	0.1975	0.7706	0.059*	
C5	1.1592 (5)	0.03798 (17)	0.6510 (2)	0.0544 (7)	
H5A	1.2626	−0.0156	0.6726	0.065*	
H5B	1.2728	0.0832	0.6261	0.065*	
C6	0.9446 (5)	0.01564 (17)	0.55255 (19)	0.0529 (7)	
H6A	0.8367	0.0685	0.5327	0.063*	
H6B	0.8352	−0.0315	0.5761	0.063*	
B	0.5724 (6)	0.3204 (2)	0.6816 (3)	0.0585 (8)	
F1	0.4655 (4)	0.37487 (13)	0.59228 (16)	0.0888 (7)	
F2	0.5357 (3)	0.23068 (11)	0.65187 (17)	0.0857 (6)	
F3	0.4510 (3)	0.33750 (12)	0.77629 (15)	0.0795 (6)	
F4	0.8355 (3)	0.33695 (11)	0.70959 (16)	0.0822 (6)	

### Atomic displacement parameters (Å^2^)

**Table:**

	*U*^11^	*U*^22^	*U*^33^	*U*^12^	*U*^13^	*U*^23^
N1	0.0566 (13)	0.0546 (13)	0.0433 (11)	0.0030 (10)	0.0081 (10)	−0.0032 (9)
C1	0.090 (2)	0.0667 (19)	0.0645 (17)	0.0065 (16)	0.0092 (16)	−0.0171 (15)
N2	0.0506 (12)	0.0452 (11)	0.0472 (10)	−0.0001 (9)	0.0144 (9)	0.0006 (9)
C2	0.0621 (17)	0.0584 (16)	0.0546 (14)	−0.0007 (13)	0.0224 (13)	0.0061 (12)
C3	0.0637 (17)	0.0472 (14)	0.0585 (15)	−0.0084 (12)	0.0233 (13)	0.0030 (12)
C4	0.0503 (14)	0.0465 (14)	0.0503 (13)	−0.0034 (11)	0.0103 (11)	0.0029 (11)
C5	0.0552 (15)	0.0531 (14)	0.0592 (15)	0.0022 (12)	0.0221 (12)	−0.0017 (12)
C6	0.0595 (16)	0.0468 (14)	0.0565 (15)	−0.0010 (11)	0.0216 (13)	−0.0020 (11)
B	0.0488 (17)	0.0561 (18)	0.075 (2)	−0.0012 (14)	0.0230 (15)	0.0105 (16)
F1	0.0884 (14)	0.0919 (13)	0.0900 (13)	0.0186 (10)	0.0262 (10)	0.0321 (11)
F2	0.0812 (12)	0.0622 (11)	0.1163 (15)	−0.0155 (9)	0.0239 (11)	−0.0018 (10)
F3	0.0691 (11)	0.0908 (13)	0.0856 (12)	0.0016 (9)	0.0330 (9)	0.0107 (9)
F4	0.0492 (10)	0.0840 (13)	0.1164 (15)	−0.0131 (9)	0.0228 (9)	0.0000 (10)

### Geometric parameters (Å, °)

**Table:**

N1—C4	1.326 (3)	C4—H4A	0.9300
N1—C2	1.369 (3)	C5—C6	1.507 (3)
N1—C1	1.465 (3)	C5—H5A	0.9700
C1—H1A	0.9600	C5—H5B	0.9700
C1—H1B	0.9600	C6—C6^i^	1.522 (4)
C1—H1C	0.9600	C6—H6A	0.9700
N2—C4	1.318 (3)	C6—H6B	0.9700
N2—C3	1.375 (3)	B—F1	1.370 (4)
N2—C5	1.472 (3)	B—F4	1.374 (3)
C2—C3	1.337 (3)	B—F2	1.381 (4)
C2—H2A	0.9300	B—F3	1.394 (4)
C3—H3A	0.9300		
			
C4—N1—C2	108.4 (2)	N1—C4—H4A	125.5
C4—N1—C1	125.3 (2)	N2—C5—C6	112.0 (2)
C2—N1—C1	126.3 (2)	N2—C5—H5A	109.2
N1—C1—H1A	109.5	C6—C5—H5A	109.2
N1—C1—H1B	109.5	N2—C5—H5B	109.2
H1A—C1—H1B	109.5	C6—C5—H5B	109.2
N1—C1—H1C	109.5	H5A—C5—H5B	107.9
H1A—C1—H1C	109.5	C5—C6—C6^i^	111.3 (3)
H1B—C1—H1C	109.5	C5—C6—H6A	109.4
C4—N2—C3	108.2 (2)	C6^i^—C6—H6A	109.4
C4—N2—C5	125.3 (2)	C5—C6—H6B	109.4
C3—N2—C5	126.4 (2)	C6^i^—C6—H6B	109.4
C3—C2—N1	107.2 (2)	H6A—C6—H6B	108.0
C3—C2—H2A	126.4	F1—B—F4	109.8 (2)
N1—C2—H2A	126.4	F1—B—F2	110.6 (3)
C2—C3—N2	107.3 (2)	F4—B—F2	108.9 (2)
C2—C3—H3A	126.3	F1—B—F3	109.2 (2)
N2—C3—H3A	126.3	F4—B—F3	109.8 (3)
N2—C4—N1	108.9 (2)	F2—B—F3	108.5 (2)
N2—C4—H4A	125.5		
			
C4—N1—C2—C3	0.4 (3)	C5—N2—C4—N1	178.3 (2)
C1—N1—C2—C3	−179.9 (2)	C2—N1—C4—N2	−0.3 (3)
N1—C2—C3—N2	−0.3 (3)	C1—N1—C4—N2	−180.0 (2)
C4—N2—C3—C2	0.2 (3)	C4—N2—C5—C6	−117.0 (3)
C5—N2—C3—C2	−178.0 (2)	C3—N2—C5—C6	60.9 (3)
C3—N2—C4—N1	0.1 (3)	N2—C5—C6—C6^i^	177.6 (2)

Symmetry codes: (i) −*x*+2, −*y*, −*z*+1.

### Hydrogen-bond geometry (Å, °)

**Table:**

*D*—H···*A*	*D*—H	H···*A*	*D*···*A*	*D*—H···*A*
C2—H2A···F1^ii^	0.93	2.50	3.328 (3)	149
C3—H3A···F3^iii^	0.93	2.51	3.398 (3)	161
C4—H4A···F2^iv^	0.93	2.46	3.272 (3)	146
C4—H4A···F3^iv^	0.93	2.45	3.326 (3)	158

Symmetry codes: (ii) *x*, −*y*+1/2, *z*+1/2; (iii) −*x*+1, *y*−1/2, −*z*+3/2; (iv) *x*+1, *y*, *z*.
